# Biogenic Fabrication of Nanoparticles With Rhododendron arboreum: A Multifunctional Platform With Antibacterial, Anti-inflammatory, and Anticancer Therapeutic Potential

**DOI:** 10.7759/cureus.92252

**Published:** 2025-09-13

**Authors:** Iram Saba, Vivek Kumar Dhiman, Susmitha Kalaichelvan, Rajasekaran Subbarayan, Ankush Chauhan, Ritesh Verma, Ahmed Ahmed Ibrahim, Khalid Mujasam Batoo, Saif Hameed

**Affiliations:** 1 Amity Institute of Biotechnology, Amity University Gurugram, Manesar, IND; 2 Centre for Herbal Pharmacology and Environmental Sustainability, Chettinad Hospital and Research Institute, Chettinad Academy of Research and Education, Kelambakkam, IND; 3 Centre for Advanced Biotherapeutics and Regenerative Medicine, Chettinad Hospital and Research Institute, Chettinad Academy of Research and Education, Kelambakkam, IND; 4 Department of Physics, Graphic Era (Deemed to be University), Dehradun, IND; 5 Department of Physics and Astronomy, King Saud University, Riyadh, SAU; 6 King Abdullah Institute for Nanotechnology, King Saud University, Riyadh, SAU

**Keywords:** antibacterial, anticancer, anti-inflammatory, green synthesis, zinc sulphide nanoparticles

## Abstract

Background: Plant-mediated nanotechnology offers a sustainable alternative to conventional nanoparticle synthesis by reducing reliance on hazardous chemicals and energy-intensive processes. Zinc sulfide nanoparticles (ZnS NPs) were selected for this study due to their wide bandgap, tunable optical properties, and promising biomedical potential compared to other semiconductor nanoparticles. *Rhododendron arboreum* flower extract, rich in flavonoids, terpenoids, and phenolic compounds, was chosen as a natural reducing and stabilizing agent to enable eco-friendly nanoparticle fabrication. We hypothesized that this phytochemical-rich extract could facilitate the green synthesis of ZnS NPs with distinctive physicochemical features and enhanced biological activity.

Methods: ZnS NPs were synthesized via a Soxhlet extract-assisted method and characterized using Fourier transform infrared spectroscopy, X-ray diffraction, scanning electron microscopy, and UV-visible spectroscopy.

Results: The nanoparticles exhibited a cubic phase structure with a crystallite size of 2.28 nm, an optical bandgap of 2.8 eV, and peak absorption at 356 nm. SEM analysis revealed irregular morphology. Biological assays demonstrated that the ZnS NPs possessed superior antibacterial activity compared to the flower extract alone, with lower minimum inhibitory concentration (MIC) and minimum bactericidal concentration (MBC) values against *Bacillus subtilis*, *Staphylococcus aureus*, *Pseudomonas aeruginosa*, and *Salmonella typhi*. The nanoparticles also exhibited significant anti-inflammatory properties by inhibiting protein denaturation of egg albumin protein at different concentrations (6.75-100 µg/mL) with inhibition values ranging from about 60% to 97%. It also showed cytotoxic and pro-apoptotic effects against HCT116 cancer cell lines.

Conclusions: These findings highlight how green synthesis using *R. arboreum* extract not only provides an eco-friendly route for ZnS NP fabrication but also yields nanoparticles with promising antibacterial, anti-inflammatory, and anticancer potential.

## Introduction

Green nanotechnology has emerged as a promising approach to nanoparticle synthesis, offering sustainable and eco-friendly alternatives to conventional chemical and physical methods that often involve hazardous reagents and high energy consumption. Among the various strategies, plant extract-mediated synthesis has gained particular attention due to its simplicity, low cost, and ability to yield stable nanoparticles through naturally occurring biomolecules such as polyphenols, flavonoids, terpenoids, and alkaloids. These phytochemicals act as both reducing and capping agents, enabling controlled fabrication of nanostructures with desirable physicochemical and biomedical properties. Nanotechnology has vast applications in the healthcare sector, including medicine delivery and illness detection [1]. As reported by Mary et al. [2], there is a potential for nanoparticles made utilizing hazardous compounds to be ingested or found on their surfaces. This has the potential to result in undesirable consequences in medical applications. Nanoparticles can be synthesized in an eco-friendly and straightforward manner using techniques inspired by natural processes [3]. Plant extract-assisted synthesis is an ecologically safe and sustainable approach for generating various nanomaterials, which has gained significant interest. Several effective endeavours have been undertaken to produce metal and metal oxide nanoparticles using plant extracts from leaves, roots, and flowers [4]. Plant extracts are considered the most suitable option for green biosynthesis, as they stabilize dispersible nanoparticles and facilitate the oxidation or reduction of metal ions during synthesis. This suitability arises from the presence of biomolecules such as polyphenols, terpenoids, phenolic acids, and alkaloids [5]. Plant extract-mediated synthesis offers a cost advantage over traditional synthesis methods by eliminating additional purification steps and using easily accessible raw materials, namely, plants. Moreover, due to the ability to easily regulate the growth and distribution of the nanoparticles, this nanoparticle production technology is both secure and environmentally friendly.

Zinc sulfide (ZnS) is a well-known semiconductor that naturally occurs in sphalerite and wurtzite crystal forms. It is widely studied for its distinctive optical and electronic properties, including a wide band gap, high refractive index, and photoluminescence, which make it useful in various technological applications [6]. Beyond its conventional role in electrical, optoelectronic, and electrochemical devices, ZnS nanoparticles (ZnS NPs) exhibit properties that differ significantly from their bulk counterparts due to their high surface area-to-volume ratio. The biomedical potential of ZnS NPs, particularly their antimicrobial activity against a range of pathogenic bacteria, is being studied. Moreover, these nanoparticles have been reported to be non-toxic to human erythrocytes, further supporting their potential for safe therapeutic use.

Among various plant sources used in green nanotechnology, *Rhododendron arboreum*, a member of the Ericaceae family, has attracted attention due to its bioactive compounds. Known for its brilliant crimson flowers, this plant has a long history of culinary and traditional medicinal use among indigenous communities of North India. Its dried flowers have been utilized to treat gastrointestinal ailments such as bleeding dysentery and diarrhea, owing to their choleretic, diuretic, anti-irritable bowel syndrome, and astringent properties. ​​​​*R. arboreum* flowers possess a distinctive combination and relatively high concentration of bioactive compounds such as polyphenols, flavonoids, terpenoids, and alkaloids compared to other commonly used plant sources [7, 8]. This enriched phytochemical profile facilitates the green synthesis of ZnS nanoparticles with desirable characteristics, including smaller particle size, narrow size distribution, high stability, and unique optical and electronic properties, making *R. arboreum* an optimal choice for this study.

ZnS NPs possess properties that make them well-suited for various applications, including biomedical imaging, drug delivery, and optoelectronic devices. Ali et al. [9] estimated the antibacterial and photocatalytic degradation activities of ZnO NPs by synthesizing them using *R. arboreum* methanolic bark extract. The mean diameter of the nanoparticles was 250 nm. Ahir et al. [10] utilized an aqueous flower extract of *R. arboreum* to synthesize nanoparticles of Dy3+-doped zinc oxide. The purpose was to evaluate the antibacterial properties and photocatalytic degradation activity of these nanoparticles. The synthesized nanoparticles had an average size of 31 nm. Similarly, Phuyal et al. [11] reported the synthesis of silver nanoparticles using aqueous leaf extract of *R. arboreum* and evaluated their metal sensing, antibacterial, and photocatalytic degradation activities. Their nanoparticles exhibited particle sizes ranging from 23 to 41 nm.

The primary objective of this research is to synthesize ZnS nanoparticles using a Soxhlet extract of *R. arboreum* flowers and to comprehensively evaluate their physicochemical and biological properties. The novelty of this study lies in the use of *R. arboreum* extract as a natural reducing and capping agent for green synthesis, coupled with detailed characterization using UV-visible spectrophotometry, FTIR, XRD, and SEM. In addition, this work systematically investigates the multifunctional bioactivities of the synthesized ZnS NPs, including antibacterial, anti-inflammatory, in vitro cell viability, and pro-apoptotic effects, providing an integrated assessment of their therapeutic potential.

## Materials and methods

Chemicals and reagents

Zinc acetate dihydrate (CH_3_COO)_2_Zn·2H_2_O was purchased from EMPLURA® (Merck, Darmstadt, Germany). Sodium sulfide flakes (extra pure, 60%) (Na₂S·xH₂O), polyethylene glycol 400 (PEG 400), and cetyltrimethylammonium bromide (CTAB) were obtained from Sisco Research Laboratories Pvt. Ltd. (SRL), Mumbai. Sodium hydroxide pellets (NaOH) and Muller Hinton Agar (MHA) were procured from HIMEDIA®, Thane. Methanol (C_2_H_5_OH) was supplied by Amichem Research Lab, Uttarakhand, and Luria Bertani broth was purchased from SRL.

Plant extract

The flowers of *R. arboreum* were collected during the spring season (March 2023) from the Solan district of Himachal Pradesh, India. The plant was identified at Nauni University in Solan, Himachal Pradesh, India (geographical coordinates: 30.91°N, 77.10°E, altitude ~1,600 m). The plant was identified, and a voucher specimen (accession no. 0698) was deposited with full herbarium records. Mature flowers were selected to ensure consistent phytochemical content, and only freshly bloomed samples were harvested.

The flowers of *R. arboreum* were subjected to a shade-drying process, followed by pulverization using an electric grinder. From 50 g of powdered flowers, the extraction yield was ~8.6% and the process was repeated in three independent batches to check reproducibility with yield variation <5%. The resultant 10 g of dried flower powder was dispersed in 100 mL of methanol and incubated in a shaker at 40°C for 48 hours. Methanol was chosen as the extraction solvent due to its superior ability to solubilize a wide range of bioactive compounds, including polyphenols and flavonoids, which are essential for the green synthesis of stable ZnS nanoparticles, compared to other solvents such as ethanol or water. The resulting supernatant was filtered through filter paper and dried in a hot air oven at 40°C for 48 hours. The dried extract was stored in a dark container at 25°C to prevent degradation of phytochemicals until further use.

Synthesis of ZnS nanoparticles

**Figure 1:**
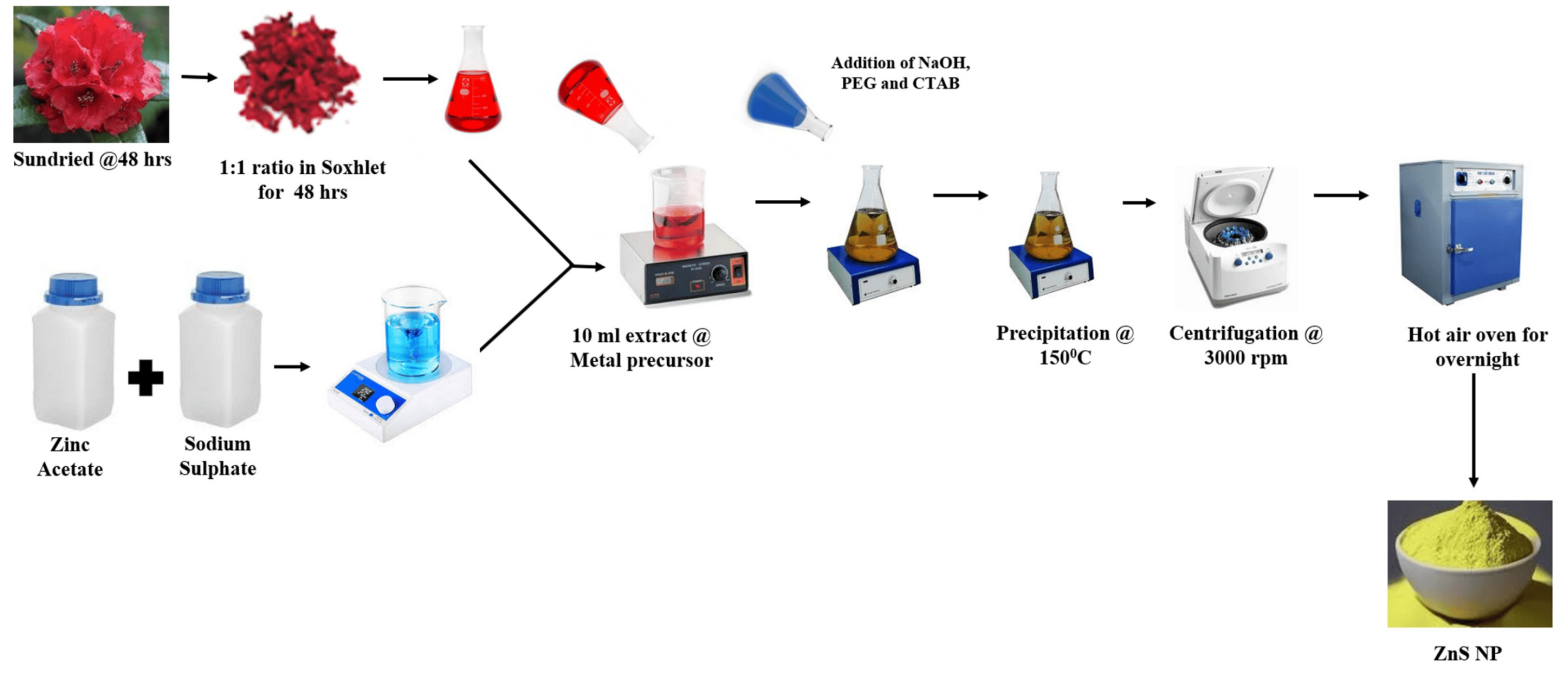
Schematic representation of the synthesis process of zinc sulfide nanoparticles (ZnS NPs) Image credit: Iram Saba

In the synthesis of ZnS NPs, an aqueous solution of 5 g of zinc acetate and 1.7 g sodium sulfide was prepared separately in 50 ml of distilled water (Figure 1).

The prepared solutions were mixed while stirring continuously. Then, 10 ml of plant extract with a 5 mg/ml concentration dissolved in methanol was added dropwise to the above solution and stirred for two hours at room temperature. Further to this solution, 5 ml of 0.05 molar NaOH aqueous solution, 5 ml of 0.05 molar CTAB aqueous solution, and 5 ml of PEG solution were added and stirred for one hour. After one hour, the temperature of the solution was increased to 150°C for one hour. The resulting precipitates were centrifuged at 10,000 rpm for 15 minutes and washed three times with distilled water, followed by ethanol to remove unreacted residues. The pellets were then dried in a hot air oven at 150 °C. This temperature was selected to ensure complete removal of moisture while preserving the structural and optical properties of the ZnS nanoparticles, as they remain thermally stable at this condition. The dried ZnS nanoparticle powder was stored in airtight amber vials at room temperature in a desiccator to prevent moisture uptake and light-induced degradation. 

Material characterization

Various analytical techniques were employed to assess the synthesized ZnS NPs. UV-visible spectrophotometry (Shimadzu UV 1800, Shimadzu Corporation, Kyoto, Japan) was utilized to measure the absorption spectrum, while dynamic light scattering (Malvern zeta sizer, Malvern Panalytical Ltd., Malvern, Worcestershire, United Kingdom) was employed to determine the hydrodynamic diameter and surface charge analysis. Fourier-transform infrared (FTIR, Bruker Corporation, Billerica, Massachusetts, USA) spectroscopy using a Bruker alpha instrument was utilized to detect distinctive chemical bonds. X-ray diffraction (XRD, Bruker Corporation, Billerica, Massachusetts, USA) patterns were analyzed to verify the phase composition of the synthesized ZnS NPs, and the structure and morphology of ZnS NPs were examined using scanning electron microscopy (SEM, Thermo Fisher Scientific, Hillsboro, Oregon, USA ).

Bacterial strain preparation

The gram-negative bacteria *Salmonella enterica* subsp. *Enterica* *serovar Typhi* (*S. typhi* ATCC 6539), *Pseudomonas aeruginosa* (*P. aeruginosa* ATCC 27853), *Bacillus subtilis* (*B. subtilis*), and gram-positive bacteria *Staphylococcus aureus* (MSSA ATCC 25923) were obtained through an acquisition from HiMedia Laboratories Pvt. Ltd. in Mumbai, India. The different strains of bacteria were cultivated in nutrient broth (HiMedia, Mumbai, India) at 37°C for 24 hours with continuous agitation at 200 rpm.

Antimicrobial activity

The minimum inhibitory concentration (MIC) of an antimicrobial agent is defined as the smallest quantity that inhibits the development of an organism in tubes or microdilutions. The MIC was used to compare the antibacterial efficacy of *Rhododendron* flower extract and green-synthesised ZnS nanoparticles. A sterile 96-well plate was infected with 100 µL of MH broth for bacterial pathogens. Test samples were initially added to the top wells of a microtiter plate at a volume of 98 µL, followed by two-fold serial dilutions across the plate. A vertical lane of wells (in triplicate) was maintained as a control. For serial dilution, 100 µL from the top wells was transferred to the subsequent wells, and the process was repeated as required. Finally, 2 µL of each pathogenic bacterial suspension was added to the wells. The infected microtiter plates were incubated with bacterial pathogens for 24 hours at 35°C. The results were thereafter monitored and recorded. Following the ascertainment of the MIC concentration for bacteria, the microbial bactericidal concentration (MBC) was determined to evaluate the presence or absence of bacterial growth on the agar plate post-incubation.

The agar-well diffusion technique was used to test the antibacterial activity of ZnS NPs that were generated by biosynthesis with some modifications. Using sterile cotton swabs, the bacterial strains were distributed on Mueller-Hinton agar (MHA) (Merck, Germany). The agar was punched with a sterile pipette tip. Each well was loaded with 10 μl of *R. arboreum* flower extract, 10 μl of ZnS NPs, 10 μl of ampicillin (positive control), and 10 μl of DMSO (negative control) individually. The whole setup was incubated at 37°C for 24 hours. A zone of inhibition was found after 24 hours of incubation.

Protein denaturation activity

The efficacy of the nanoparticles to inhibit the denaturation of egg albumin protein was examined by following Velidandi et al. [12] with some modifications. The standard inhibitory test process includes 112 μl of PBS buffer, 8 μl of egg white solution, and the subsequent addition of 80 μl of NPs and plant extract at increasing volumes (6.75, 12.5, 25, 50, and 100 μl) from a stock solution with a concentration of 100 mg/mL, respectively. This results in final concentrations of 6.75, 12.5, 25, 50, and 100 μg/ml. The final volume of the reaction was adjusted to 200 μl by adding buffer to all the wells of the 96-well plate. The plate was incubated for 15 minutes at 37°C. Subsequently, the 96-well plate was subjected to heat at 80°C for five minutes in a water bath, after which the plate was allowed to cool. The absorbance of the samples was measured at a wavelength of 660 nm. The reaction tube containing aspirin was used as the positive control. The percentage of protein denaturation inhibition was calculated using the formula: (A_c_−A_s_)/A_c_×100, where A_c_ is the absorbance of the control and A_s_ is the absorbance of the sample.

In vitro cell viability assay of ZnS NPs in the HCT116 cell line

The cytotoxicity of ZnS NPs was measured using a colorimetric assay system that utilized tetrazolium salt, specifically 3-[4,5-dimethylthiazol- 2-yl]-2,5-diphenyl tetrazolium bromide MTT. The HCT116 cell line was subjected to different concentrations of ZnS NPs for 24 hours. The cytotoxicity of the treatment was assessed using the MTT assay. The calculation for determining the percentage of cell viability is as follows: The cell viability (%) was calculated using the formula: mean optical density of the sample divided by the mean optical density of the blank × 100.

In vitro Alamar Blue assay for the assessment of the anticancer effect of ZnS NPs

The viability and cytotoxicity activity of HCT116 cells were tested in the presence of reported ZnS NPs. HCT116 cells were seeded into 96-well plates at a concentration of 1 × 105 cells/ml and incubated for 24 hours in a standard incubator. After 24 hours, cells were provided with fresh medium containing different concentrations of ZnS NPs (1, 5, 10, and 25 µg/mL) or without ZnS NPs. After 24 hours of culture, Alamar Blue was introduced directly into culture media at a final concentration of 10%, and optical densities were measured after an additional 24-hour and 48-hour period.

Apoptosis analysis

Phase contrast images were captured, and viable and dead cells were recorded based on cellular morphology. After 24-hour post-ZnS NPs treatments, 6-diamidino-2-phenylindole dihydrochloride (DAPI) staining was used to study the nuclear fragmentation within the tested group of cells. Control versus different doses of ZnS NPs (after 24-hour treatment) were stained with DAPI (300 nM) and subjected to fluorescent imaging by Nikon Eclipse Ti2 Fluorescence inverted microscope (Nikon Corporation, Melville, New York).

Statistical analysis

Statistical analyses were performed using GraphPad Prism 9. One-way ANOVA followed by Tukey’s post-hoc test was employed to examine differences in the effects of ZnS NPs and the methanolic extract of *R. arboreum* flowers between the control and treatment groups. All data are presented as mean ± standard deviation (SD). Sample sizes for each experiment were triplicate for antimicrobial assays and n = 3 independent experiments for cell assays. A significance threshold of p < 0.0001 was used to indicate statistically significant differences.

## Results

UV-visible spectrophotometry

**Figure 2:**
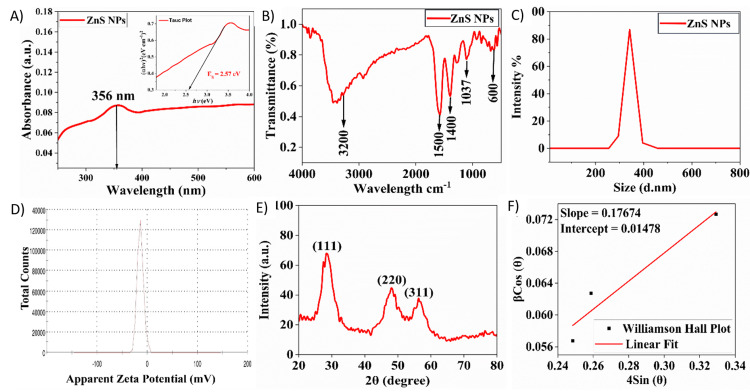
A) UV-visible spectra and band gap energy of ZnS NPs, B) FTIR spectrum of ZnS NPs, C) DLS analysis of ZnS NPs, D) zeta analysis of ZnS NPs, E) XRD pattern, and F) W-H plot of ZnS NPs ZnS NPs: zinc sulfide nanoparticles, FTIR: Fourier transform infrared spectroscopy, DLS: dynamic light scattering, XRD: X-ray diffraction, W-H: Williamson-Hall

Figure 2A presents the absorption spectra. This is used to investigate the optical band gap of ZnS NPs. The fundamental absorption correlates with electron excitation and calculates the energy band gap. The UV-vis absorbance results were used to create a "Tauc plot" [(hν) vs (αhν)2] of the prepared nanoparticles. The optical band gap values were calculated using the Tauc plots.

FTIR analysis

Figure 2B shows the FTIR spectrum of ZnS NPs synthesised from *R. arboreum* flowers and illustrates distinct absorption peaks, offering valuable insights into the nanoparticles' chemical composition and bonding characteristics. 

Dynamic light scattering and zeta analysis

Figure 2C-2D shows the dynamic light scattering (DLS) size of 333.9 nm and a zeta potential of -13.8 mV, providing valuable information about the physical properties and stability of ZnS NPs derived from *R. arboreum* flowers.

X-ray diffraction

The XRD pattern seen in Figure 2E provides evidence that the synthesized ZnS NPs were effectively produced utilizing the green synthesis approach and had a crystalline structure.

Morphological analysis

**Figure 3:**
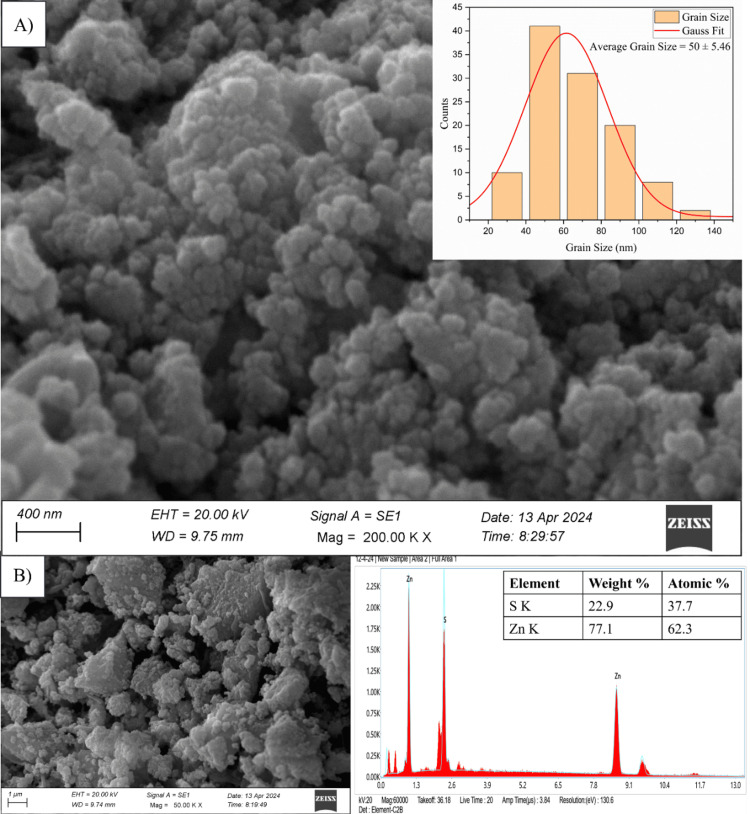
A) SEM image and grain size distribution, B) EDX of ZnS NPs ZnS NPs: zinc sulfide nanoparticles, SEM: scanning electron microscopy, EDX: energy-dispersive X-ray spectroscopy

The SEM micrograph and grain size distribution of the ZnS NPs are shown in Figure 3A. Here, the small crystallites were observed to aggregate spontaneously, forming larger grains with an irregular morphology. The SEM image demonstrates the process of smaller particles coming together to create bigger grains. The average size of these grains is 50 ± 5.46 nm. The EDX demonstrates a consistent distribution of elements and high elemental purity, specifically regarding zinc and sulphide concentrations in the Zn K and S K regions (Figure 3B). The weight percentages of Zn and S were determined to be 77.1% and 22.9%, respectively. In addition, their atomic weight percentages were discovered to be 62.3% and 37.7%, respectively.

Antimicrobial activity

MIC and MBC Evaluation

ZnS-NPs showed lower MIC than *Rhododendron* flower extract (Table 1). This indicates that ZnS-NPs exhibited higher antibacterial activity against *Bacillus subtilis*, *S. aureus*, *Pseudomonas aeruginosa*, and *S. typhi* than *Rhododendron* flower extract. However, *Rhododendron* extract showed good antibacterial activity but was ineffective against the *S. typhi* strain.

**Table 1:** MIC and MBC of Rhododendron extract and green synthesized ZnS-NPs. MIC: minimum inhibitory concentration, MBC: minimum bactericidal concentration, ZnS-NPs: zinc sulfide nanoparticles

	MIC		MBC	
Microbial strains	*Rhododendron* extract (mg/mL)	ZnS-NPs (µg/mL)	*Rhododendron* extract (mg/mL)	ZnS-NPs (µg/mL)
Bacillus subtilis	4	200	8	>400
Staphylococcus aureus	8	200	16	>400
Pseudomonas aeruginosa	16	800	32	>1000
Salmonella typhi	-	600	-	>800

Agar-well diffusion method

**Figure 4:**
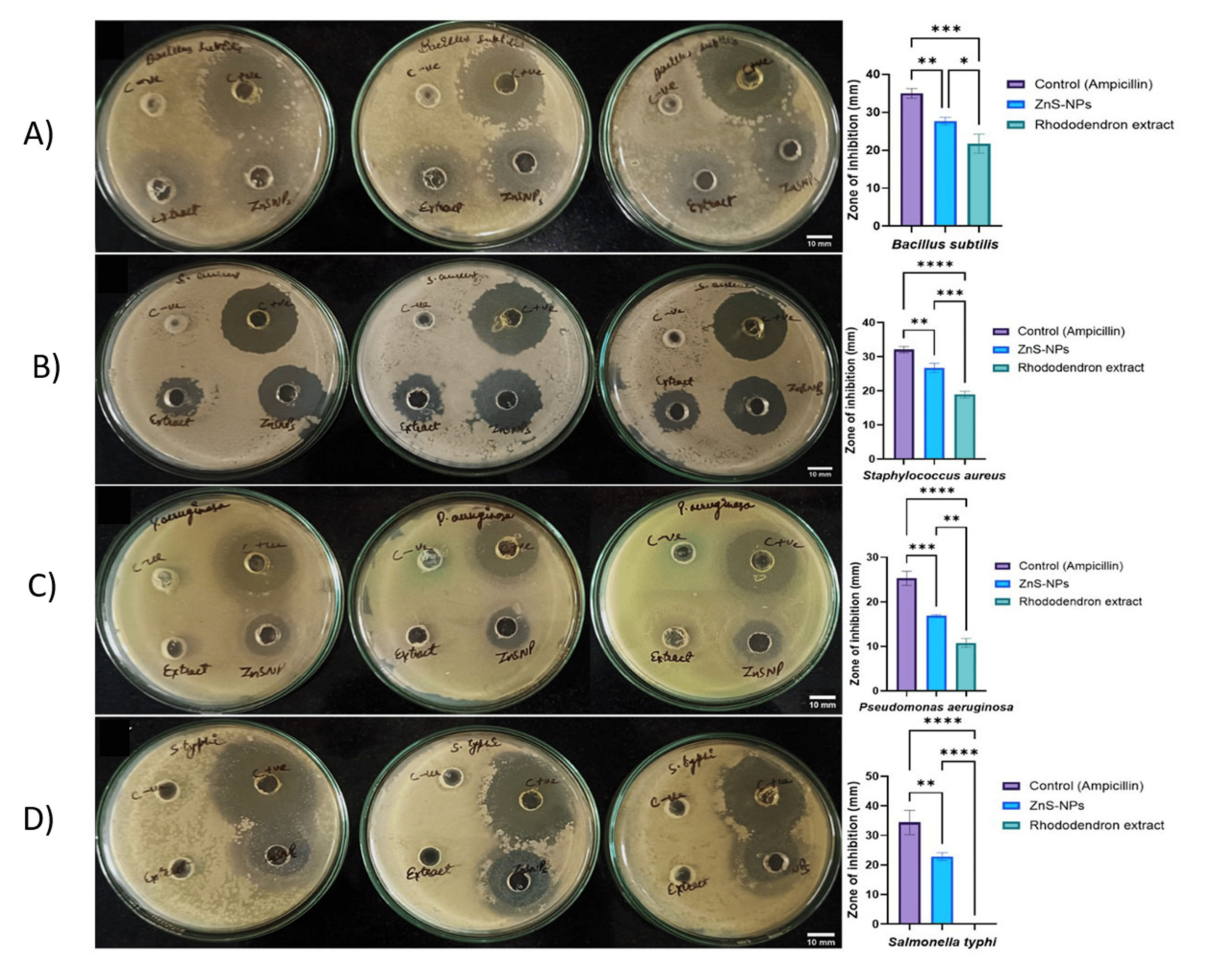
Antibacterial activity and zone of inhibition of ZnS NPs against pathogenic bacteria: A) Bacillus subtilis, B) Staphylococcus aureus (MSSA ATCC 25923), C) Pseudomonas aeruginosa (ATCC 27853), D) Salmonella typhi (ATCC 6539) using Rhododendron arboreum flower extract (50 mg/mL), ZnS NPs (1 mg/mL), ampicillin (+ve control), and DMSO (negative control). Every value signifies the average ± standard deviation of three duplicates. Values are presented as mean ± SD (n = 4). **** p < 0.0001, and ns p-value is no significant value (negative control). ZnS NPs: zinc sulfide nanoparticles, DMSO: dimethyl sulfoxide

The antimicrobial activity of ZnS nanoparticles was evaluated using the agar-well diffusion assay against targeted pathogens (Figure 4A-4D). Compared to the extract, ZnS NPs showed a larger zone of inhibition against pathogenic bacteria. The zones of inhibition for *B. subtilis*, *S. aureus*, *P. aeruginosa*, and *S. typhi* were 27.68 ± 1.00, 26.77 ± 1.35, 16.86 ± 0.24, and 22.86 ± 1.36 mm (Table 2), respectively.

**Table 2:** Zone of inhibition of ZnS NPs and Rhododendron arboreum flower extract for various bacterial strains (mean ± SD). ZnS NPs: zinc sulfide nanoparticles, DMSO: dimethyl sulfoxide

Strains	ZnS (ZOI in mm)	Positive control (ZOI in mm)	Negative control (DMSO)	*Rhododendron arboreum* flower extract (ZOI in mm)
Bacillus subtilis	27.68 ± 1.00	35.01 ± 1.25	0	21.76 ± 2.51
Staphylococcus aureus	26.77 ± 1.35	32.10 ± 0.93	0	18.91 ± 0.96
Pseudomonas aeruginosa	16.86 ± 0.24	25.3 ± 1.50	0	10.8 ± 1.00
Salmonella typhi	22.86 ± 1.30	34.9 ± 4.11	0	-

Protein denaturation assay

**Figure 5:**
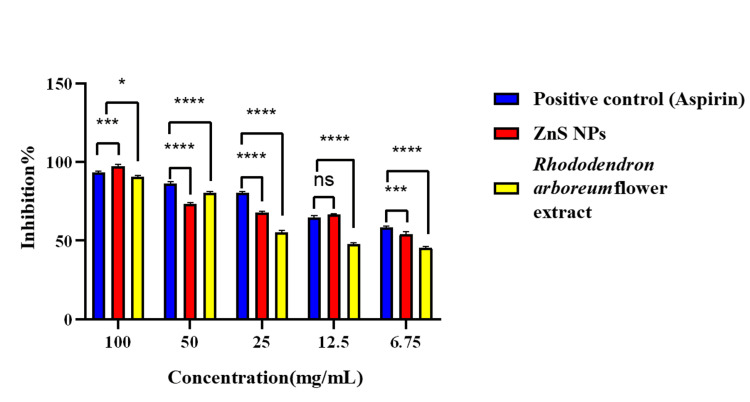
Anti-inflammatory activity of ZnS NPs and plant extract versus positive control. Values are presented as mean ± SD (n = 3). ****p < 0.0001, ***p < 0.001, and ns p-value is insignificant. ZnS NPs: zinc sulfide nanoparticles

The study examined the efficacy of ZnS NPs in preventing the denaturation of egg albumin protein under heat stress conditions at different concentrations. The examined NPs have shown inhibitory action that varies depending on the concentration (Figure 5). The anti-inflammatory analysis findings show a dose-dependent relationship, where the anti-inflammatory effect increases as the quantity of green-produced nanoparticles and fractions rises from 6.75 to 100 µg/mL.

Assessment of cytotoxicity and pro-apoptotic effects of ZnS NPs in HCT116 cells

**Figure 6:**
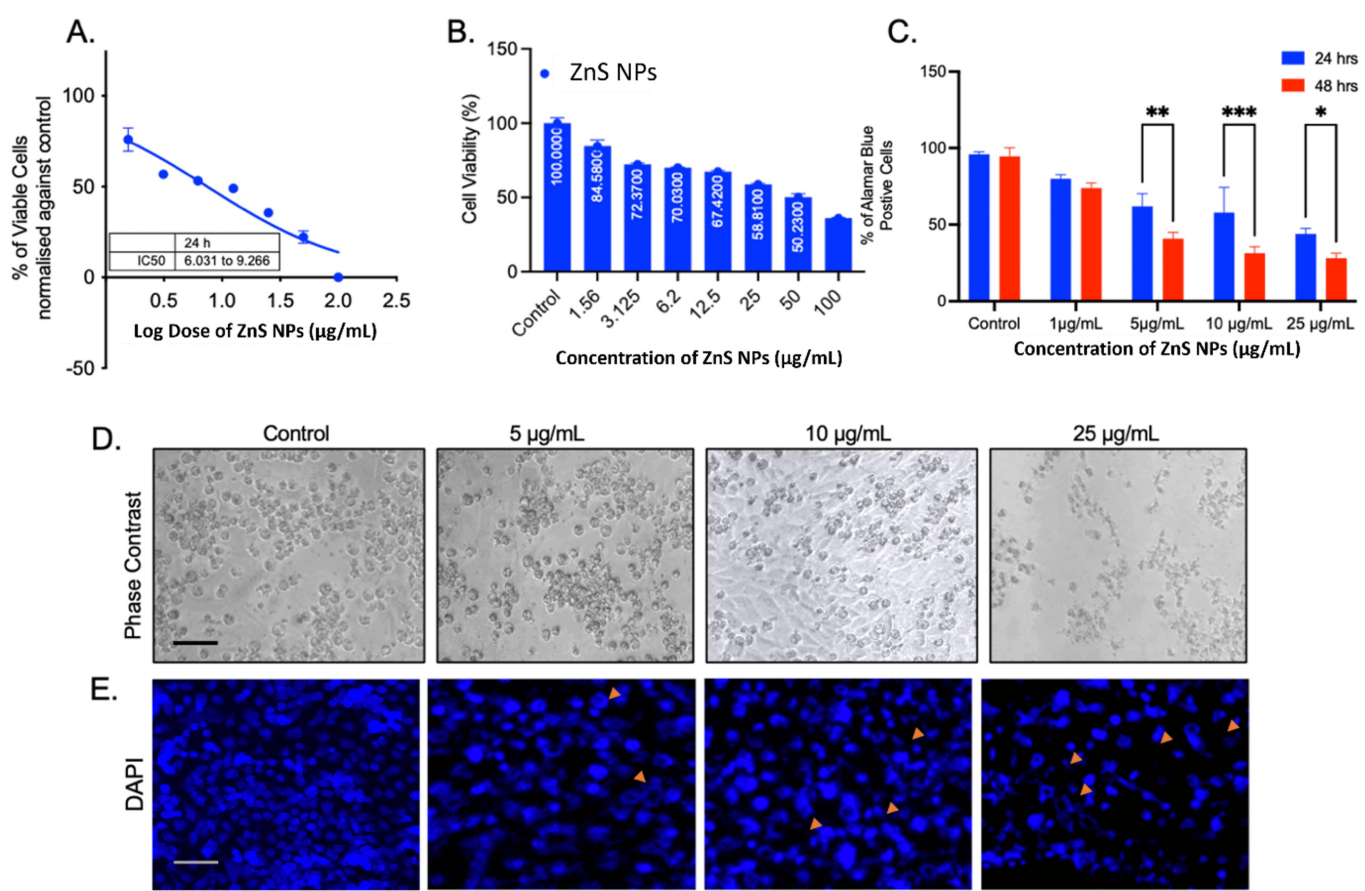
The MTT assay demonstrates the non-linear regression analysis of ZnS NPs and displays the IC50 value (A). Furthermore, (B) illustrates the dose-dependent effect of ZnS NPs on the viability of HCT116 cells. (C) The Alamar Blue assays demonstrate the effectiveness of ZnS NPs treatments in combating cancer. The phase contrast image displays the morphological assessment of ZnS NPs-treated cells (D). The DAPI images show the presence of nuclear fragmentation following ZnS NP treatments at low to higher concentrations of 0, 12.5, 25, and 100 μg/mL (E). All the images were captured at 200x magnification with a 20-micrometer scale bar. ZnS NPs: zinc sulfide nanoparticles, DAPI: 4′,6-diamidino-2-phenylindole

Cell viability was assessed in the HCT116 colon cancer cell line after exposure to ZnS NPs at concentrations ranging from 0 to 100 μg/ml. The analyses revealed a gradual decrease in cell viability in the HCT116 cell line. Linear regression analysis was conducted to determine the 50% inhibitory concentration (IC50) (Figures 6A-6B). According to the study, it is recommended to use a concentration of 6-10 mM for ZnS NPs in in vitro experiments. Furthermore, the Alamar Blue assay was employed to assess the potential anti-cancer properties of ZnS NPs in HCT116 cells. The results indicate that the 24-hour and 48-hour treatments of ZnS NPs effectively suppress the growth of cancer cells, with inhibition observed at concentrations ranging from 1 mg/ml to 25 mg/ml (Figure 6C). This finding highlights the significant potential of ZnS NPs in effectively inhibiting the proliferation of cancer cells. In addition, the apoptotic study showed that the ZnS NPs treatments significantly increased the number of cells undergoing apoptosis (Figures 6D-6E). The ZnS nanoparticles demonstrated dose-dependent cytotoxicity and pro-apoptotic effects in HCT116 cells, indicating potential anti-cancer activity.

## Discussion

The present study demonstrates the eco-friendly synthesis of nanoparticles using *R. arboreum* flower extract, which simultaneously acted as a reducing and stabilizing agent. The biosynthesized particles were confirmed to possess a cubic crystalline structure, nanoscale dimensions, and distinctive optical properties, signifying the effectiveness of the plant extract in mediating nanoparticle formation. Importantly, the synthesized particles exhibited enhanced antibacterial, anti-inflammatory, and anticancer activities compared to the crude extract, highlighting the biomedical potential of this green nanotechnology approach. Our findings not only expand the repertoire of medicinal plants that can be harnessed for sustainable nanomaterial production but also emphasize the added value of utilizing phytochemical-rich extracts to confer therapeutic functionalities.

In the UV-visible spectrophotometry, Tangents were applied to the Tauc plot's 'x-axis' to estimate the band-gap value. ZnS NPs exhibit a band gap of approximately 2.8 electron volts (eV), indicating their semiconducting nature. This band gap value signifies the energy difference between the valence band, where electrons are tightly bound to atoms, and the conduction band, where electrons can move freely and conduct electricity under the influence of an external energy source. Such a moderate band gap renders ZnS suitable for various applications, including optoelectronics, solar cells, and a catalyst.

The FTIR analysis spectrum reveals characteristic peaks corresponding to functional groups in the ZnS NPs and the organic compounds derived from the *R. arboreum* flower. For instance, the peak at 600 cm^-1^ signifies Zn-S stretching vibrations, indicating the presence of Zn-S bonds within the nanoparticles [13]. The peak observed at 1037 cm⁻¹ is also attributed to C-O stretching vibrations originating from organic compounds found in the *R. arboreum* flower, such as polysaccharides or flavonoids. Peaks in the 1400 cm⁻¹ to 1500 cm⁻¹ indicate C-H bending vibrations from organic molecules in the flower extract used during nanoparticle synthesis. Moreover, the peak at 3200 cm⁻¹ corresponds to O-H stretching vibrations, which may arise from hydroxyl groups in the flower extract or surface hydroxyl groups on the ZnS NPs.

The DLS size of 333.9 nm in Figure 2C suggests that the nanoparticles are polydispersed and have a relatively sizeable hydrodynamic size, which could be attributed to agglomeration or the presence of surface coatings or stabilizers. Figure 2E represents the surface charge of nanoparticles in a colloidal dispersion. A negative zeta potential indicates that the nanoparticles have an overall negative surface charge, suggesting potential electrostatic repulsion between particles and a certain degree of stability against aggregation. However, a zeta potential of -13.8 mV indicates a relatively low surface charge, possibly rendering the nanoparticles susceptible to agglomeration. The formation of larger grains, despite the zeta potential suggesting electrostatic repulsion, can be explained by the complex interactions among nanoparticles [14]. Although the zeta potential of -13.8 mV suggests that the ZnS NPs have a moderate negative charge, leading to electrostatic repulsion, other factors can dominate and drive agglomeration. The relatively low zeta potential value indicates that the repulsive forces may not be sufficient to overcome attractive forces such as van der Waals interactions, hydrophobic forces, or hydrogen bonding. In addition, the large hydrodynamic diameter (333.9 nm) obtained from DLS suggests that the nanoparticles may be forming loose clusters, which can still exhibit electrostatic repulsion on their surface while being held together by weaker attractive forces. This combination of repulsive and attractive forces can form larger grains, resolving the apparent contradiction [15].

The X-ray diffraction peaks at 28.6°, 48.1°, and 56.2° belong to (111), (220), and (311) (hkl) planes of ZnS crystal, respectively, which are in good agreement with the cubic phase JCPDS file no. 80-0020. Sharp crystalline peaks confirm the crystallinity of the green-synthesized nanoparticles [16]. Through the use of the Scherrer formula, the average crystallite size was determined. The calculated crystallite size for ZnS was 2.28 nm. The William-Hall equation was utilized to ascertain the lattice strain (ε) [17]. The strain generated within the crystal is a result of crystal imperfections. The strain was calculated from the slope of the plot between β cos θ along the y-axis and 4sin θ along the x-axis corresponding to each peak of the XRD pattern, as given in Figure 2F. Furthermore, the XRD pattern of the synthesised nanoparticles has no impurity phase, revealing that the NaOH, PEG, and CTAB were added as stabiliser, reducing agent, and capping agent, respectively [17].

The enhanced antibacterial activity of ZnS NPs observed in the MIC and MBC evaluations can be attributed to their bactericidal interactions, likely mediated by antimicrobial phenolics and other bioactive compounds present in *R. arboreum* flowers. Specifically, the MIC values of ZnS NPs ranged from 4000 to 16,000 µg/mL, compared to 200 to 800 µg/mL for the flower extract alone. The MBC values ranged from 8000 to 32,000 µg/mL for ZnS NPs, compared to 400 to 1000 µg/mL for the extract. These results demonstrate the superior efficacy of the nanoparticles in inhibiting bacterial growth and are consistent with previously reported studies [18].

**Figure 7:**
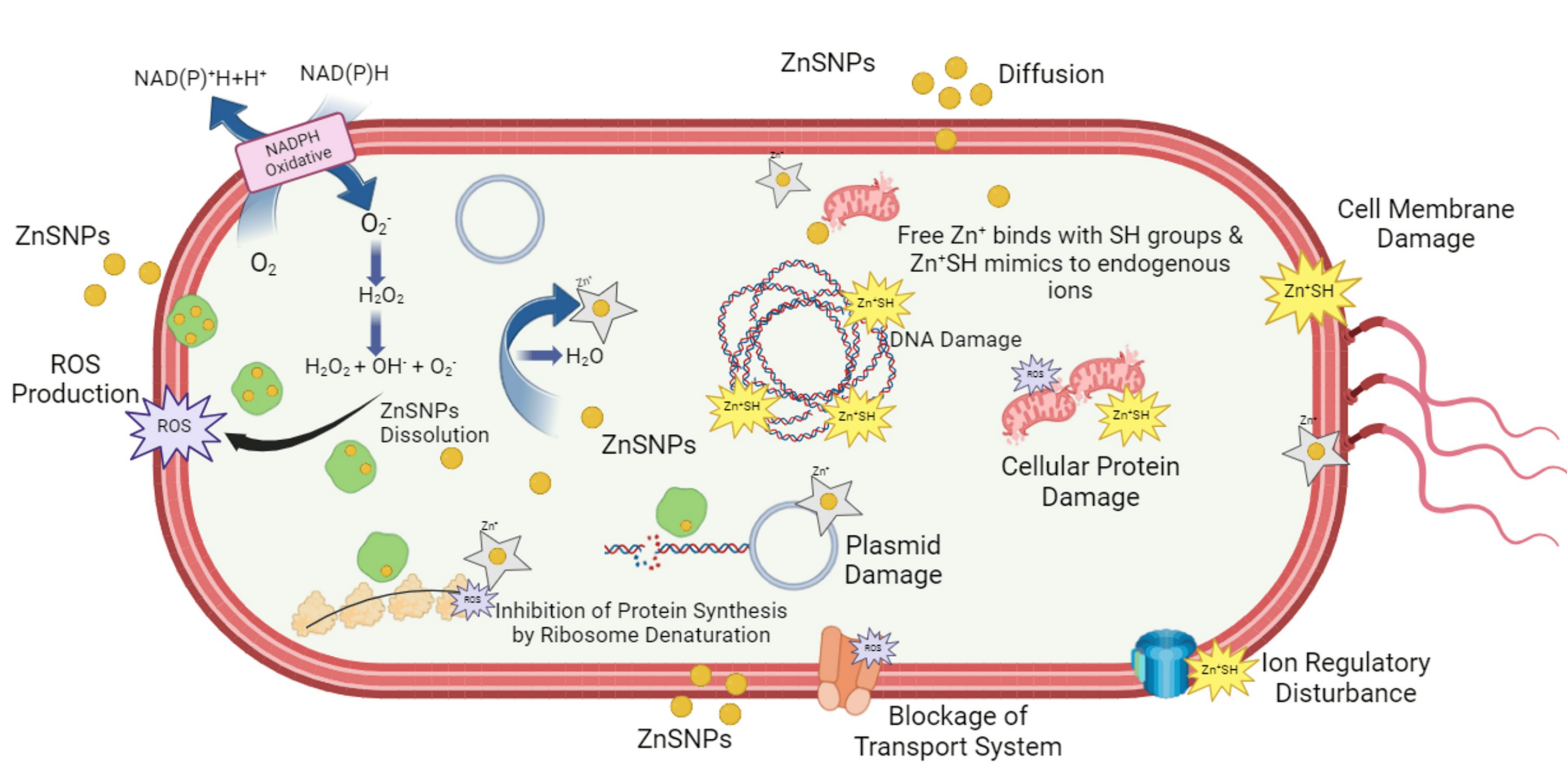
Schematic depiction of the antibacterial activity Image credit: Ankush Chauhan

In the agar diffusion analysis, the characteristic of the bacterial surface is a negative charge, which creates an electromagnetic attraction of ZnS NPs with the lipopolysaccharide layer. A positive charge of zinc ions surrounds the bacterial layer, embedding themselves into the lipid bilayer and permeating into cells, all triggering oxidation events [19]. Zinc ions stimulate electrostatic contact between the negatively charged cell membrane of bacteria and the positively charged nanoparticles [20]. Moreover, the high surface-to-volume ratio of the nanoparticles contributes to their enhanced activity against various microorganisms [21]. ZnS NPs interact with cellular components through electron-donating molecules, such as thiols, amides, polysaccharides, indoles, and hydroxyls, and interfere with the activity of biological enzymes, DNA and RNA replication, and halt cellular processes. This initiates a series of related events, generating ROS, such as superoxide, hydroxyl radicals, and hydrogen peroxide. Zinc ions form complexes with proteins and enzymes, so impeding their function and causing a disruption in the cellular redox equilibrium [22]. Figure 7 shows the mechanism of antibacterial activity.

The figure illustrates that the liberation of zinc ions induces a modification in the reactivity of thiol groups in bacterial protein, which suppresses DNA replication in bacteria [23]. This interaction leads to pore formation in bacterial cell walls, increasing permeability and causing subsequent cell death [24].

In the protein denaturation assay, the protein denaturation occurs mainly in individuals afflicted with arthritic and inflammatory conditions [25]. Protein denaturation is an extensively researched phenomenon that often occurs under stressful conditions, including exposure to organic solvents, strong acids or bases, high salt concentrations, or heat [26]. Proteins may lose their biological roles and structural stability when they undergo denaturation [27]. Nanoparticles synthesized using plant extracts possess potent phytochemicals with anti-inflammatory effects, making them a promising option for medication development in the battle against inflammatory illnesses [28].

This study has several limitations that should be acknowledged. First, although *R. arboreum* flower extract was employed as a natural reducing and capping agent, the inclusion of CTAB and PEG as stabilizers indicates that the synthesis represents a partially green approach rather than a fully plant-mediated method. While these agents were necessary to maintain colloidal stability and regulate nanoparticle growth, future works should explore plant-derived or biodegradable stabilizers to improve sustainability. Second, the observed discrepancy between crystallite size determined by XRD and hydrodynamic size measured by DLS likely reflects nanoparticle aggregation, solvation layers, or methodological differences, which requires further systematic investigation. Third, the biological evaluation, while covering antibacterial, anti-inflammatory, and anticancer potential, was limited to four bacterial strains and a single cancer cell line. This restricts the ability to draw conclusions regarding safety and selectivity. Lastly, this study did not include a comparative analysis with ZnS NPs produced via conventional chemical synthesis, which would be important to contextualize the added value of the green synthesis approach. Addressing these limitations in future work will provide stronger evidence to support the biomedical potential of plant-mediated ZnS NPs.

## Conclusions

This study demonstrated the successful synthesis of ZnS NPs using *R. arboreum* flower extract, supported by physicochemical characterization confirming their cubic crystal structure and optical properties. The ZnS NPs displayed antibacterial activity superior to the extract alone, as well as measurable anti-inflammatory and cytotoxic effects in vitro. While these findings highlight the potential biomedical relevance of ZnS NPs, the synthesis should be regarded as partially green due to the use of stabilizers, and the observed biological effects remain preliminary. Future work should include in vivo validation, mechanistic investigations, and comparative studies with conventionally synthesized ZnS.
